# A novel magnetically controlled bioreactor for ex vivo expansion of NK-92 cells

**DOI:** 10.1186/s40643-022-00537-z

**Published:** 2022-05-03

**Authors:** Yangyang Liu, Qihao Sun, Mengyang Hao, Wen‑Song Tan, Haibo Cai

**Affiliations:** State Key Laboratory of Bioreactor Engineering, East China University of Science and Technology, Shanghai, 200237 China

**Keywords:** NK-92 cells, Bioreactor, Magnetic field, Ex vivo expansion

## Abstract

The application of natural killer (NK) cells as potential antitumor effector cells appears to be valuable for immunotherapies. However, the clinical use of NK cells is limited because the technical difficulties associated with mass production NK cells at sufficiently high numbers represents a great challenge. Ex vivo expansion of NK cells is a key technology for cell therapy. Bioreactor systems can generate homogeneous culture condition and modulate the environmental and biochemical cues. In this study, a novel magnetically controlled bioreactor was developed for supporting NK cells ex vivo expansion. Using synthetic magnetic beads, the stirring device of the magnetically controlled bioreactor generated reduced shearing force. The intermittent magnetic field was applied for magnetic beads movement to homogenize the culture system. NK-92 cells were cultured in the magnetically controlled bioreactor and the expansion and function of expanded cells were investigated on day 8. The results showed that the expansion of NK-92 cells in the bioreactor was 67.71 ± 10.60-fold, which was significantly higher than that of the T25 culture flask (*P* < 0.05). Moreover, the proportions of CD3^−^CD56^+^ cells and cell killing activity of expanded cells in the bioreactor did not reveal any differences compared to T25 flasks. Taken together, this study demonstrated the possibility of magnetically controlled bioreactor as a potent strategy in NK cells production for facilitating cancer immunotherapy.

**Graphical Abstract:**

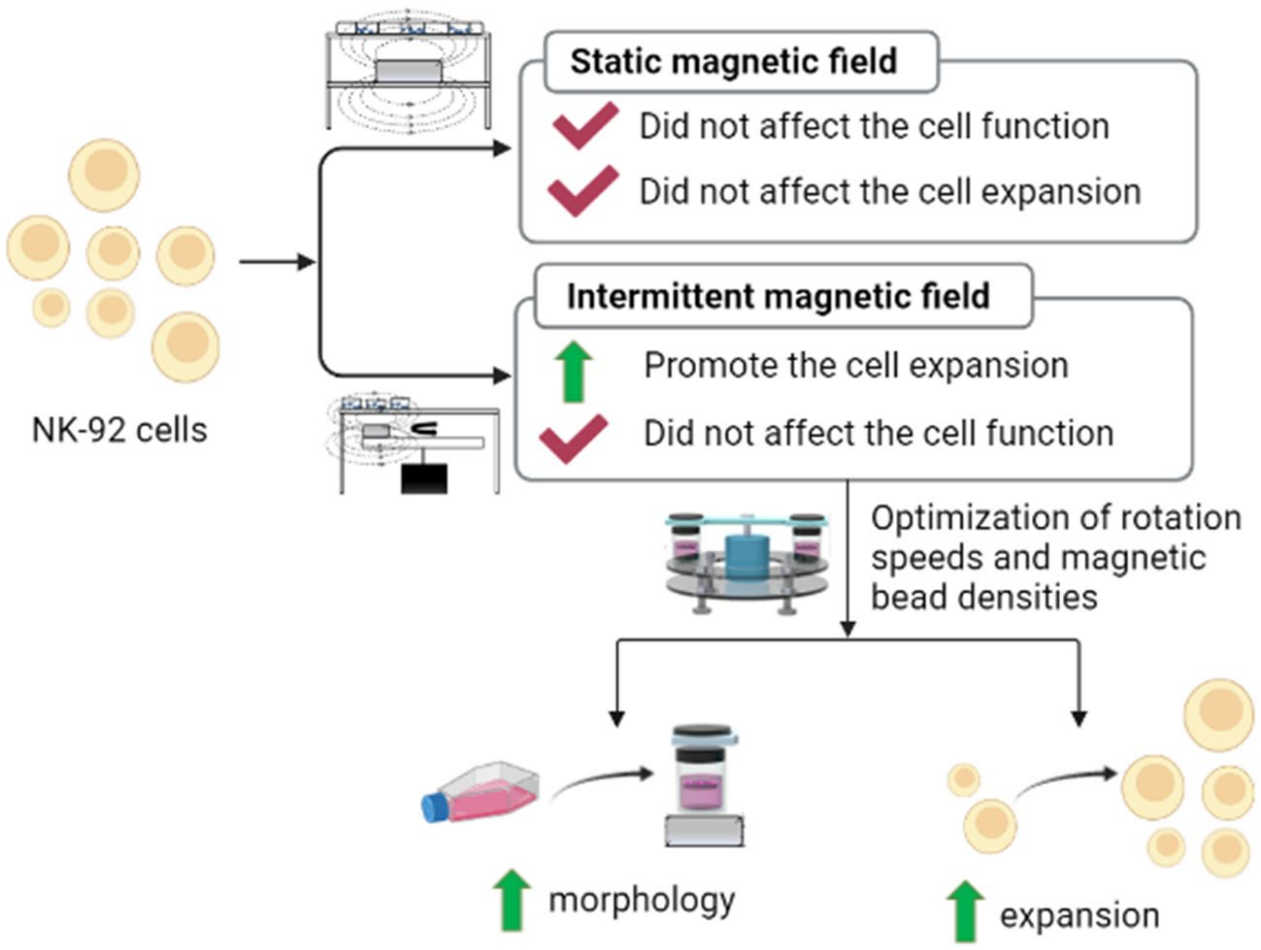

## Introduction

Natural killer (NK) cells are innate immune cells that lyse tumor cells in a non-major histocompatibility complex (MHC) restricted manner, which makes it a promising candidate for adoptive immunotherapy (Wang et al. 2017; Ljunggren and Kärre 1990; Wang et al. 2017). However, there are technical challenges in obtaining sufficient numbers of functionally active NK cells from a patient's blood since they represent only 10% of the lymphocytes and are often dysfunctional (Klingemann et al. 2016; Zhang et al. 2019). NK-92 is a highly activated NK cell line which was originally established from a non-Hodgkin’s lymphoma. And NK-92 is homogenous cell populations, which has a typical NK profile mainly CD3^−^CD56^+^ population, compared to NK cells (Gong et al. 1994; Luetke-Eversloh et al. 2013; Verheyden and Demanet 2008). They can be expanded substantially in the presence of interleukin-2 (IL-2) without the need for allogeneic feeder cells, which makes them suitable for cancer immunotherapy (Wang et al. 2017; Suck et al. 2016). Since NK-92 is readily available from a current (c)-GMP-compliant master cell bank, predictable and reproducible expansion of an extensively characterized potent NK cell agent holds great promise for clinical application (Suck et al. 2016).

NK cells need to be expanded before clinical infusion to meet the dosage demand. The optimization of ex vivo expansion processes depends on a variety of factors, and culture system is a key factor to overcome the technical obstacle. The large-scale production of cells is mainly achieved in bioreactors. Bioreactor systems have the advantages of providing a homogenous culture environment, real-time monitoring and control of cell culture process, which enables transparent and controllable. Bioreactors create a consistent culture condition and guarantee homogeneous supply of cultured cells with nutrients and gasses for efficient expansion strategy.

For immune cell expansion of bioreactor systems such as the shake flasks, rotating wall vessels, WAVE bioreactor, and stirring bioreactor have been investigated (Meng et al. 2018; Zhang et al. 2018; Ou et al. 2019; Kaiser et al. 2015). In stirring bioreactors, mixing of culture medium that exposed the cells with impeller cause to exert the fluid shear stress. However, immune cells do not have cell wall and are sensitive to shear force. Then different types of impellers produce different shear forces during operation, and spherical agitation produces less shear force than impeller type agitation (Collignon et al. 2010; Hosseinizand et al. 2016; Mckee and Chaudhry 2017). Rotating bioreactors generate a low-shear stress culture environment, allowing to partially overcome the limitations of stirred tank devices. However, the complexity of the technological solutions adopted for rotation make these devices not easily scalable and unsuitable for continuous medium replacement and real-time monitoring (Rodrigues et al. 2011). Sutlu et al. expand large numbers of clinical-grade NK cells in a Wave bioreactor without feeder cells. Expanded cells consisted 38% CD3^−^CD56^+^ NK cells and have a higher cytotoxic capacity than cells expanded in flask (Sutlu et al. 2010). Meander type bioreactors which is the directed laminar flow of medium and minimize cell stress, achieved extensive expansion of highly pure (> 85%) and potent anticancer active NK cells (Bröker et al. 2019). Therefore, an optimal bioreactor system is needed for the expansion of effector cells.

At present novel equipment is continuously developed to keep shear forces in large-scale bioreactors low, and magnetically controlled bioreactor should represent a potential alternative. Magnetically controlled bioreactor is mainly composed of magnetic material and magnetic fields. The spherical mixer is controlled by non-contact mode to blend the culture condition. Through the movement of the spherical magnetic material, the concentration gradient of the substance is eliminated and the culture condition is evenly homogeneous. At the same time, the shear force is lower than the shear force generated by the traditional stirring impeller, avoiding the mechanical damage to cells. Magnetic field is divided into weak magnetic fields (< 1mT), medium magnetic fields (1mT–1 T), strong magnetic fields (1–20 T) and ultrastrong magnetic fields (> 20 T), depending on the magnetic field intensity. Furthermore, according to the type of magnetic field, it can be divided into static magnetic field, alternating magnetic field, pulsed magnetic field and rotating magnetic field. The effect of magnetic field on cell growth and function has been investigated, and the type and intensity of magnetic field act as the main parameters. Although lots of ex vivo and in vivo experiments have been studied, the effects of magnetic field on biological systems, experimental coherence among different studies is still lacking. Early study showed that 0.8 Hz, 120 mT maximum uniform pulsed magnetic fields exposure increased NK cytotoxic activity (De Seze et al. 1993). However, 50 Hz, 2 mT magnetic field suppressed NK cell activity in guinea pigs (Canseven et al. 2006). The viability and cytotoxicity of human NK cell line were enhanced when cultured under 0.4-T static magnetic field. Lin et al. (2019) observed that strong static magnetic field (10 T) decreased naive peripheral blood T cells, while no difference in number of NK cell subpopulation was found (Onodera et al. 2003). It is indicated that magnetic fields of different types, field intensity or frequencies can lead to diverse results. Magnetic fields within reasonable range could be applied to support ex vivo expansion of NK cells in bioreactor and maintain function integrity.

In this study, the magnetically controlled bioreactor was developed for ex vivo expansion of NK-92 cells. The effects of magnetic field on the growth and activity of NK-92 cells were investigated. Further, the optimal culture system parameters were determined based on cell growth. The expansion folds, immunophenotype, and killing activity of expanded NK-92 cells were evaluated to validate effect of the culture system. The magnetically controlled bioreactor supported ~ 70-fold NK-92 cell expansion within 8 days of culture without feeder cells. This study provides a promising platform for ex vivo expansion of immune cells.

## Materials and methods

### Magnetic field exposure device

**Fig. 1:**
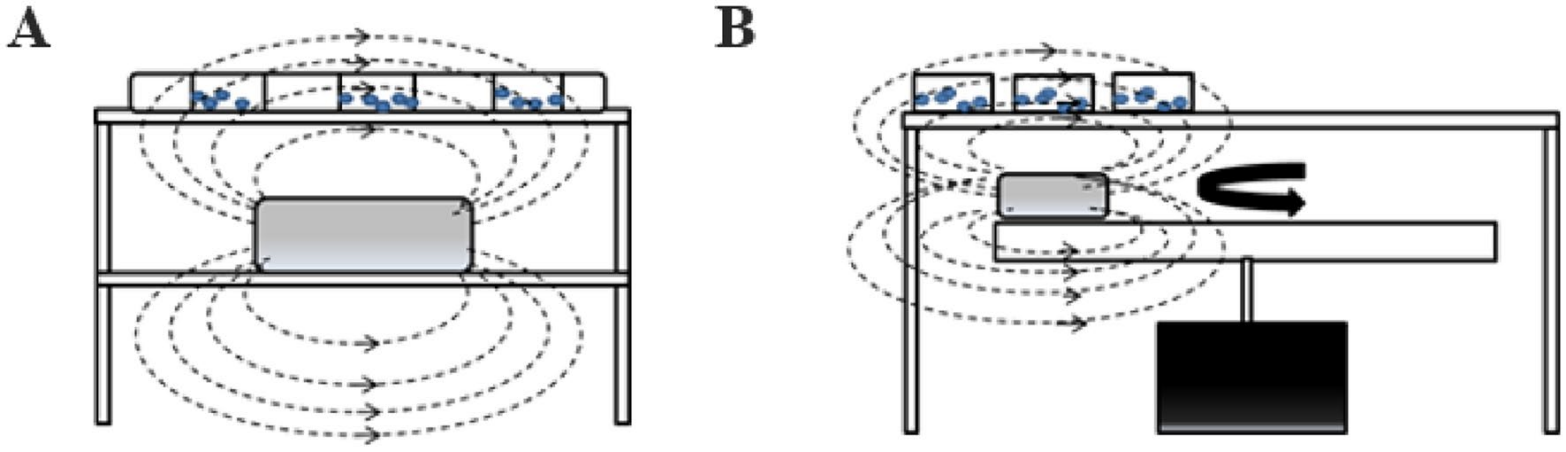
Device for generating different types of magnetic fields. **A** Static magnetic field device. **B** Intermittent magnetic field device

Static magnetic field and intermittent magnetic field were set for cell exposure. A static magnetic field was produced by placing a permanent neodymium magnet on the lower layer of the bracket, where different static magnetic field intensity was generated at different positions on the upper layer of the bracket (Fig. 1A). Intensity of the obtained static magnetic field were determined by Gauss meter at about 10 mT, 50 mT and 100 mT, respectively.

The intermittent magnetic field was produced by fixing the magnet on the crossbar, and the well plate was placed on the bracket above the magnet (Fig. 1B). The rotation of the crossbar drives the magnet to make a circular motion, and the magnet passes under the well plate at intervals to form different intermittent magnetic field around the orifice plate. Intensity of the obtained intermittent magnetic field was determined at about 10 mT, 50 mT and 100 mT, respectively, and the intermittent frequency of the intermittent magnetic field was 0.5 Hz. During the experiment, the magnetic field device was introduced to the CO_2_ incubator.

### Preparation of magnetic beads

The magnetic beads were prepared by a physical cross-linking method. Simply put, 2.5 g of sodium alginate was fully dissolved in 100 ml 1.0% (v/v) acetic acid. 3 g of Fe_3_O_4_ nanoparticles were mixed to the solution. The Fe_3_O_4_ nanoparticles were purchased from Aladdin. The magnetic beads were obtained by pipetting the solution to 300 mmol/l of calcium chloride and placing it on a 130 r/min shaker for 30 min. Then, the magnetic beads were transferred to a chitosan solution of 5 g per liter and placed on a shaker at 130 revolutions per minute for 1 h to obtain sodium alginate–chitosan magnetic beads. The obtained magnetic beads were repeatedly washed three times with ultrapure water to remove unreacted chitosan on the surface, and finally soaked in a medium for use.

### Cell culture

NK-92 cells are from American Type Culture Collection (ATCC). NK-92 cells were seeded at 2 × 10^5^ cells/ml and cultured in serum-free medium T009 (Bioengine, Shanghai) containing 1000 U/ml of IL-2 (PeproTech, USA). The cells were passaged every 2 days. Fresh medium and 1000 U/ml IL-2 were added to maintain the cell density at 2 × 10^5^ cells/ml for a total 8 days of culture. Similarly, NK-92 cells were seeded into the bioreactor at 2 × 10^5^ cells/ml while adding the appropriate amount of magnetic beads according to the volume.

### Cell viability, proliferation rate, and cell counting

Cell viability was determined by trypan blue staining. For cell counting, supernatants in the bioreactor and T25 flasks were mixed sufficiently and collected every other day. The kinetics of cell growth was calculated according to the following equation:

1 $${\text{Specific growth rate:}}\,\mu { = }\frac{\ln N2 - \ln N1}{{t2 - t1}},$$

where *μ* was the specific growth rate of cells, *N*1 was the number of cells at the time point of *t*1, *N*2 was the number of cells at the time point of *t*2.

### Flow cytometry

Cells were harvested from the magnetically controlled bioreactor and T25 flasks on day 8. A total of 1 × 10^6^ cells were washed with phosphate buffered saline (PBS) and resuspended. Then, cells were incubated with FITC-conjugated anti-human CD3 antibody and PE-conjugated anti-human CD56 antibody (BD, USA) for 30 min at 4 °C in dark. Samples were analyzed by flow cytometer (FACS Aria I, BD, USA) to determine the proportions of CD3^−^CD56^+^ cells in total cell population.

### Physiological function assays of expanded NK-92 cells

Physiological function of expanded NK-92 cells was determined by their cytotoxic capacity on tumor cells. Expanded NK-92 cells were collected as effector cells (E) and K562 cells, from Shanghai cell bank of Chinese Academy of Sciences, as target cells (T). K562 cells (5 × 10^4^ cells/ml) were cultured in DMEM (Gibco, USA) with 10% serum (Hyclone, USA). 50 μL density adjusted effector cells and target cells suspension were added into the same well of 96-well culture dish, used as the experimental group. At the same time, 50 μL density adjusted effector cells and target cells suspension were added into different wells, and then 50 μL medium was added, respectively, used as the control group. Cells were incubated for 24 h in 5% CO_2_ cell incubator at 37 °C. Finally, 10 μL CCK8 reagent was added to each well, and incubated in 5% CO_2_ cell incubator at 37 °C for 1–4 h. The OD value at 450 nm was detected and expressed by OD_E_, OD_T_ and OD_ET_, respectively. The mortality of K562 cells was calculated according to the following formula as the killing activity of effector cells:

$${\text{Killing activity}} = \left( {1 - \frac{{{\text{OD}}_{{{\text{ET}}}} - {\text{OD}}_{{\text{E}}} }}{{{\text{OD}}_{{\text{T}}} }}} \right) \times 100{\text{\% }}{.}$$

### Statistical analysis

Data were presented as mean ± standard error. Student’s *t*-test was applied to evaluate the significance of differences. *P* < 0.05 was considered as statistically significant.

## Results and discussion

### Magnetically controlled bioreactor development

**Fig. 2:**
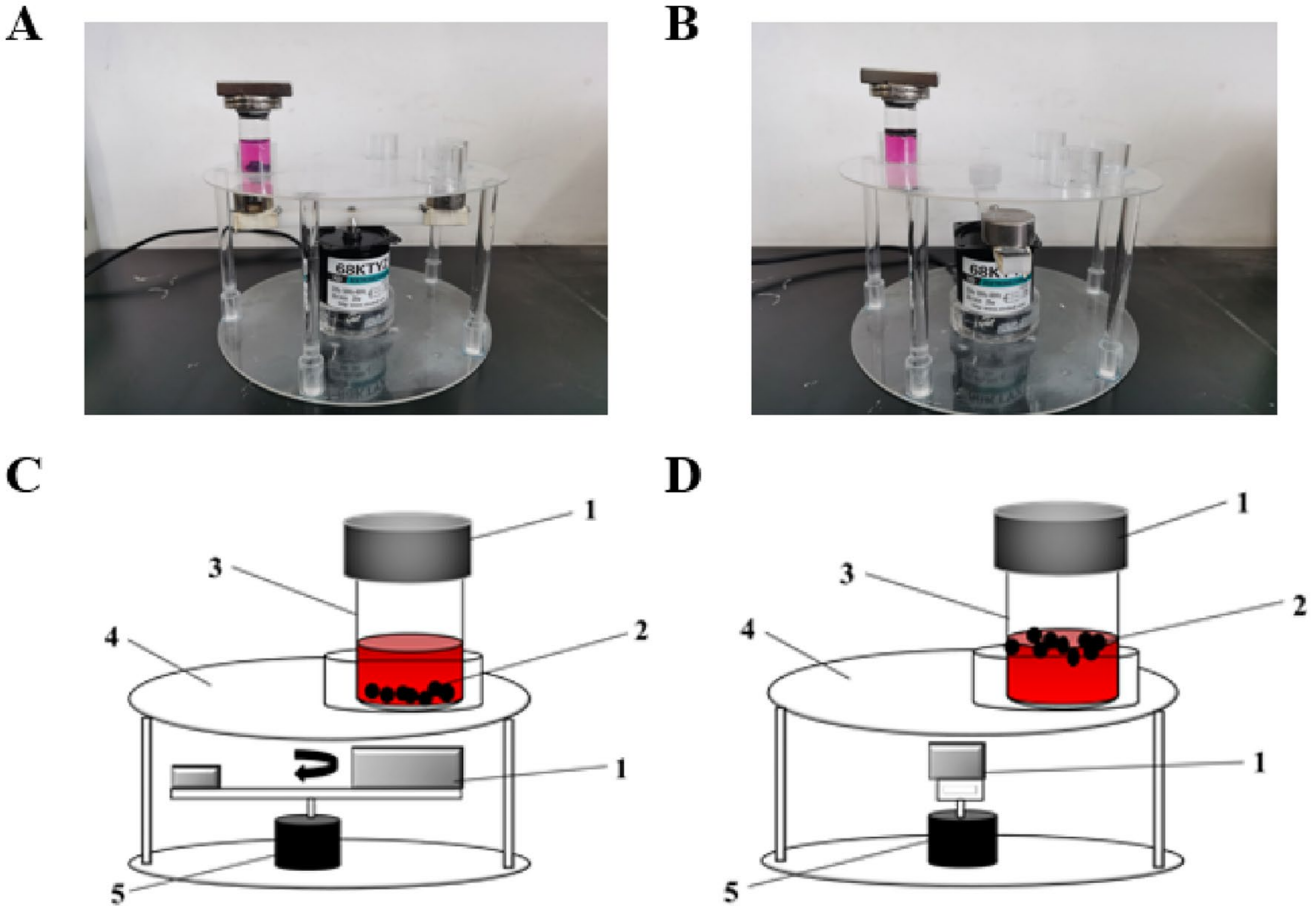
**A**, **B** Reality images of a magnetically controlled bioreactor. **C**, **D** Schematic diagram of a magnetically controlled bioreactor. 1, magnet; 2, magnetic hydrogel beads; 3, reaction vessel; 4, bracket; 5, drive machine

The magnetically controlled bioreactor including reactor holder, reaction vessel, drive machine and magnets was designed (Fig. 2). The reactor bracket was divided into upper and lower layers, wherein a groove was designed in the middle of the lower layer to fix the driving machine, and a plurality of grooves were arranged on the upper layer to fix the reaction container. In addition, the magnetic beads were filled within the reaction vessel. The magnets consisted of two parts, a part of which was a ring magnet and placed on the top of the reaction vessel to attract the magnetic beads to suspend on the surface of the liquid. The other part was fixed on the crossbar of the link driver and performed circular motion with the rotation of the crossbar. When the magnet moved to the bottom of the reaction vessel, the magnetic beads are attracted to the bottom. The magnetic beads resuspend when the magnet left. The frequency of the magnetic beads floating is closely related to the rotational speed of the crossbar. For example, under the condition of 10 r/min, the magnetic ball moves every 3 s to complete a round of floating up and down, and the magnetic beads can be instantly adsorbed to one end and hover there. Under the set experimental conditions, it can be directly observed that the magnetic ball has enough time to move from one end to the other end. By floating the magnetic beads up and down, culture system was homogenized.

### Static magnetic field did not affect the expansion and function of NK-92 cells

**Fig. 3:**
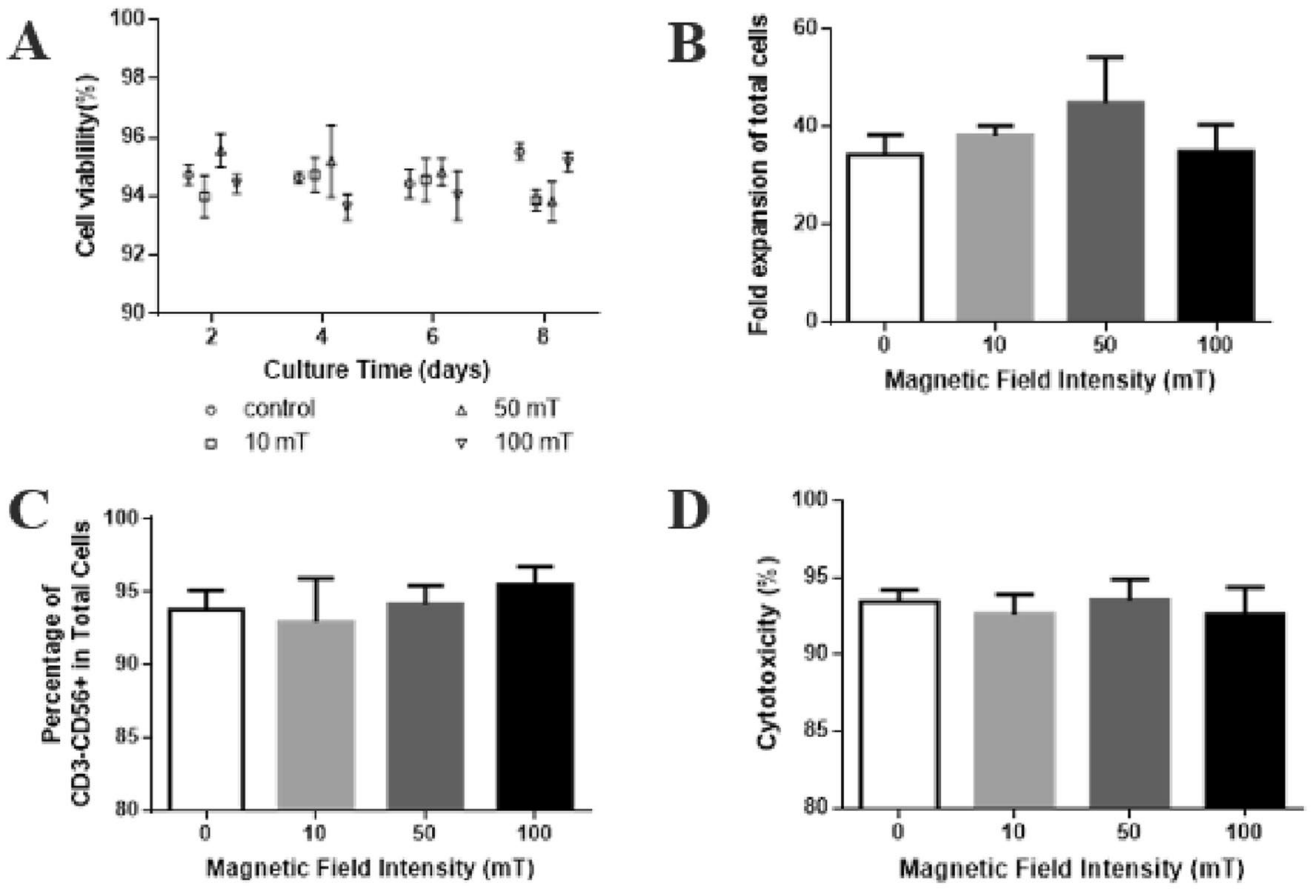
Effect of static magnetic field on NK-92 cells. **A** Fold expansion of total cells. **B** Cell viability. **C** Percentage of CD3^−^CD56^+^cells. **D** Cytotoxicity of the expanded NK-92 cells. *n* = 3

Studies had shown that regardless of the magnetic induction intensity, SMFs alone had no lethal effect on cell survival under normal culture conditions, and had no significant effect on genetic toxicity. Also, the growth rate and cell cycle distribution of most cells were not affected by SMFs (Miyakoshi 2005). We assessed the effect of magnetic field on the cell growth, phenotype and cytolytic function of NK-92 cells by culturing NK-92 cells without magnetic field (control) or 10 mT, 50 mT and 100 mT static magnetic field, respectively. The results showed that the viability of NK-92 cells remained above 92% in both the control and magnetic field groups during the 8-day culture process (Fig. 3A). The expansion folds of NK-92 cells in static magnetic field were 38.26 ± 1.63, 44.96 ± 9.32 and 35.02 ± 5.58, which showed no significant difference from that of control (34.33 ± 4.11) (Fig. 3B). Similarly, there was no difference in CD3^−^CD56^+^ cell population and the cytotoxicity toward the K562 cells between static magnetic field-expanded and control NK-92 cells (Fig. 3C, D). These results indicated that static magnetic field showed no apparent effect on the ex vivo expansion and function of NK-92 cells.

Although the research on NK cells at the cellular level point out that exposure to a 400 mT constant magnetic field increased the viability of NK92-MI cells and their ability to kill K562 tumor cells was also improved (Lin et al. 2019), the possible reason was that the ratio of E:T and the magnetic field strength both were somewhat different. Overall, the medium static magnetic field mentioned here at least supported the maintenance of the viabilities, proliferation and cytotoxicity of NK cells without biological toxicity.

### Intermittent magnetic field promoted the expansion and function of NK-92 cells

**Fig. 4:**
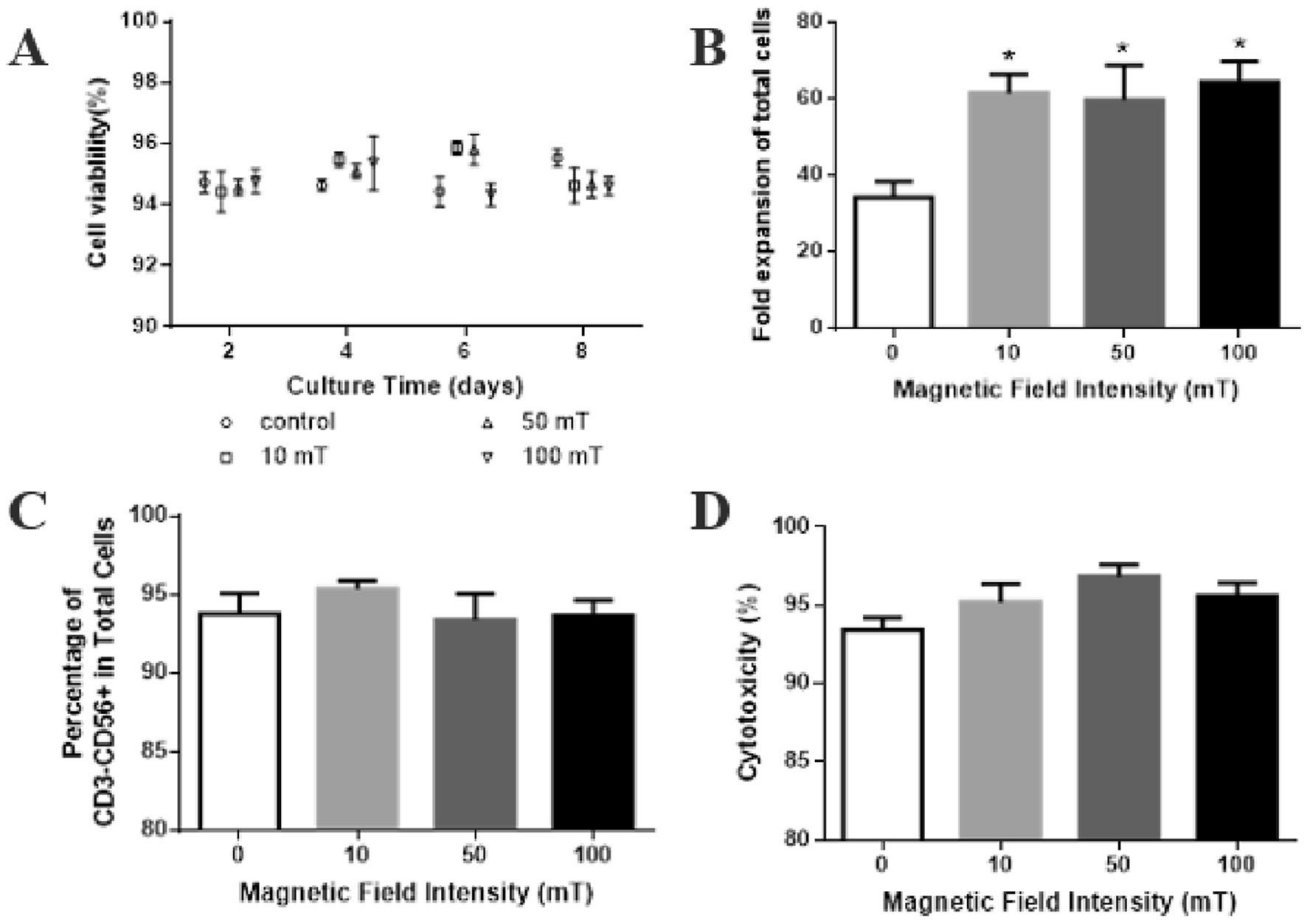
Effect of intermittent magnetic field on NK-92 cells NK-92 cells under. **A** Cell viability. **B** Fold expansion of total cells. **C** Percentage of CD3^−^CD56^+^cells. **D** Cytotoxicity of expanded NK-92 cells. *n* = 3, *P < 0.05

It was known that the biological effects of magnetic fields can be influenced by the magnetic field types, strength, frequency, treatment time and other parameters, all of which contribute to the mixed results of biological effects of magnetic field in the literature (Zhang et al. 2017). Previous studies on the effect of MFs on NK cells were focused on individual level (House and Mccormick 2000; Onodera et al. 2003; Gobba et al. 2009), and there were few studies on the influence of direct exposure to magnetic fields on NK cells. To investigate the effect of intermittent magnetic fields on the growth of NK-92 cells, the intensities were set to 10 mT, 50 mT and 100 mT with 30 Hz intermittent frequency. Similarly, cell viabilities of the NK-92 cells from magnetic field and the control group were both above 92% at all time points (Fig. 4A). Notably, the expansion folds of NK-92 cells cultured in the intermittent magnetic field were 61.55 ± 4.93, 59.77 ± 9.07 and 64.46 ± 5.42, respectively, which is significantly higher than 34.33 ± 4.11 in control (Fig. 4B). No differences in the frequency of CD3^−^CD56^+^ cells and cytotoxicity activity were detected in NK-92 cells expanded under intermittent magnetic field compared to control cells (Fig. 4C, D). These findings suggested that intermittent magnetic fields improved NK-92 cell expansion while maintaining the frequency of CD3^−^CD56^+^ cells and cytotoxicity.

This is the first study to assess the effects of intermittent magnetic fields on the survival, expansion and function of NK cells. The mechanism of the magnetic field affecting cells mainly includes the generation of induced currents, causing ion distribution and movement, and changing the membrane potential, thereby changing the permeability of the cell membrane (Dini et al. 2005). Although the specific mechanism had not been explored, these results indicated that the magnetic field strength and magnetic field type in this study can be safely applied to the in vitro expansion of NK cells, which provides an alternative for large-scale expansion of NK cells.

### Optimization of culture conditions in magnetically controlled bioreactor

**Fig. 5:**
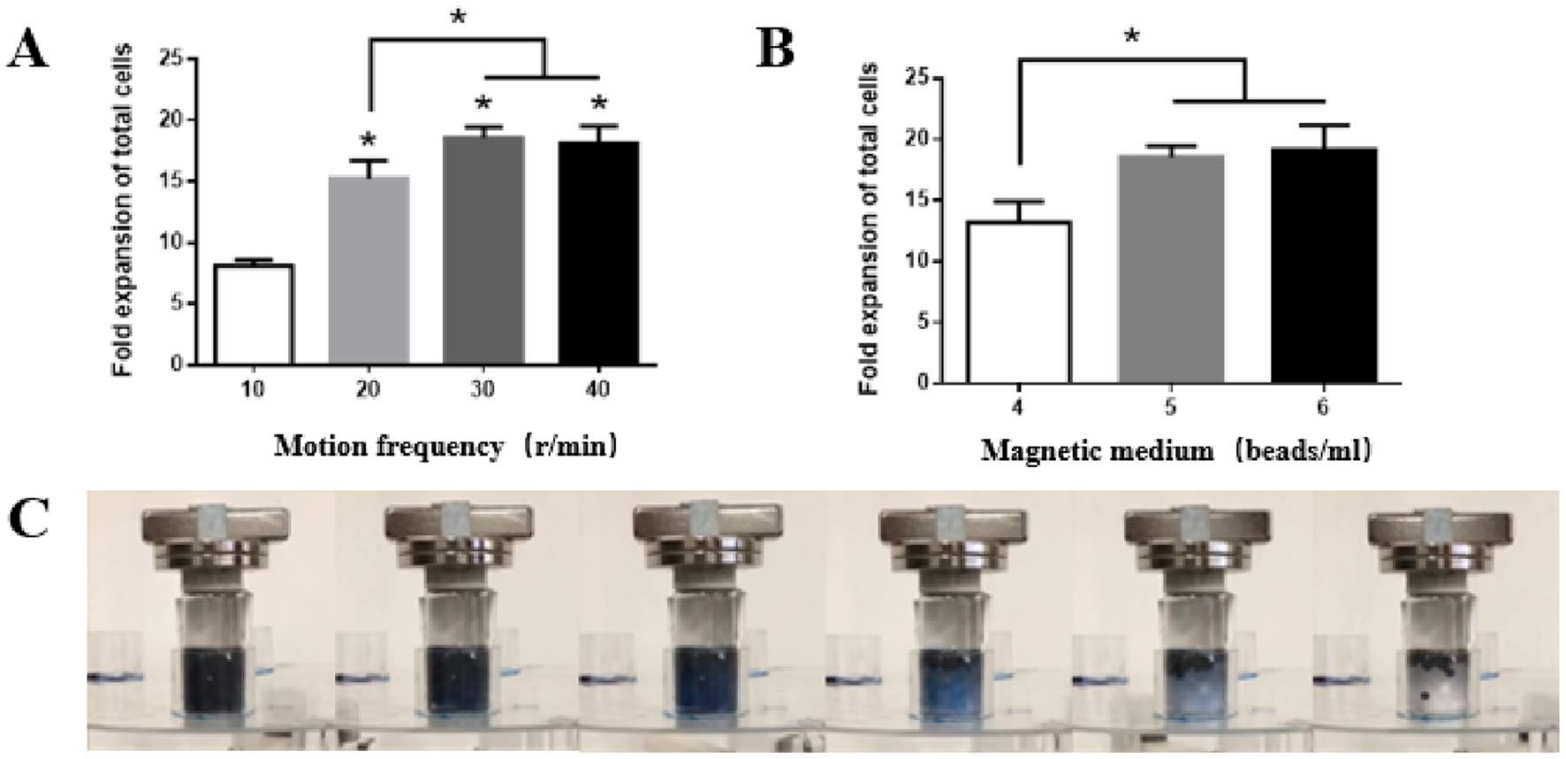
Optimization of culture conditions in magnetically controlled bioreactor. **A** NK-92 cell expansion fold at different rotation speeds. **B** NK-92 cell expansion fold at different magnetic beads densities. **C** Images of the effect of mixing medium in the rotational frequency of the crossbar

The optimized culture conditions of the magnetically controlled bioreactor for the cells were studied by setting different rotation speeds and different magnetic bead densities. The rotation speeds and magnetic bead densities were key parameters that influenced the property of magnetically controlled bioreactor. K562 cells were used as model and cultured with different rotation speeds and magnetic bead densities. We confirmed that the expansion of total cells was enhanced while the rotational speeds above 10 rpm (Fig. 5A). At 30 rpm, the expansion fold was 18.59 ± 0.74, similar to 40 rpm, but significantly higher than the expansion fold of rotation speed at 20 r/min. Given that cells were incapable of suspending in the culture system at low rotational speeds, the optimal number of magnetic beads in the magnetically controlled bioreactor need further determined. The expansion folds of K562 cells were 18.59 ± 0.74 and 19.17 ± 1.63 at 5 beads/ml and 6 beads/ml of magnetic bead, which was significantly higher than the expansion with magnetic bead density of 4 beads/ml (Fig. 5B). This result may be due to the fact that the magnetic bead density is too low to effectively suspend the cells in the culture system. This result suggested that rotation speeds of 30 r/min and magnetic beads density of 5 per ml are recommended for the cultivation of cells in the magnetically controlled bioreactor.

The homogeneity of liquid mixing within the reaction vessel was evaluated by color fading test. Results showed that the entire culture system completely mixed while the crossbar rotates five times (Fig. 5C). The results indicated that the magnetic beads inside the magnetron bioreactor can effectively mix the culture system.

### Characterization of flask and bioreactor for NK-92 cells expansion

**Fig. 6:**
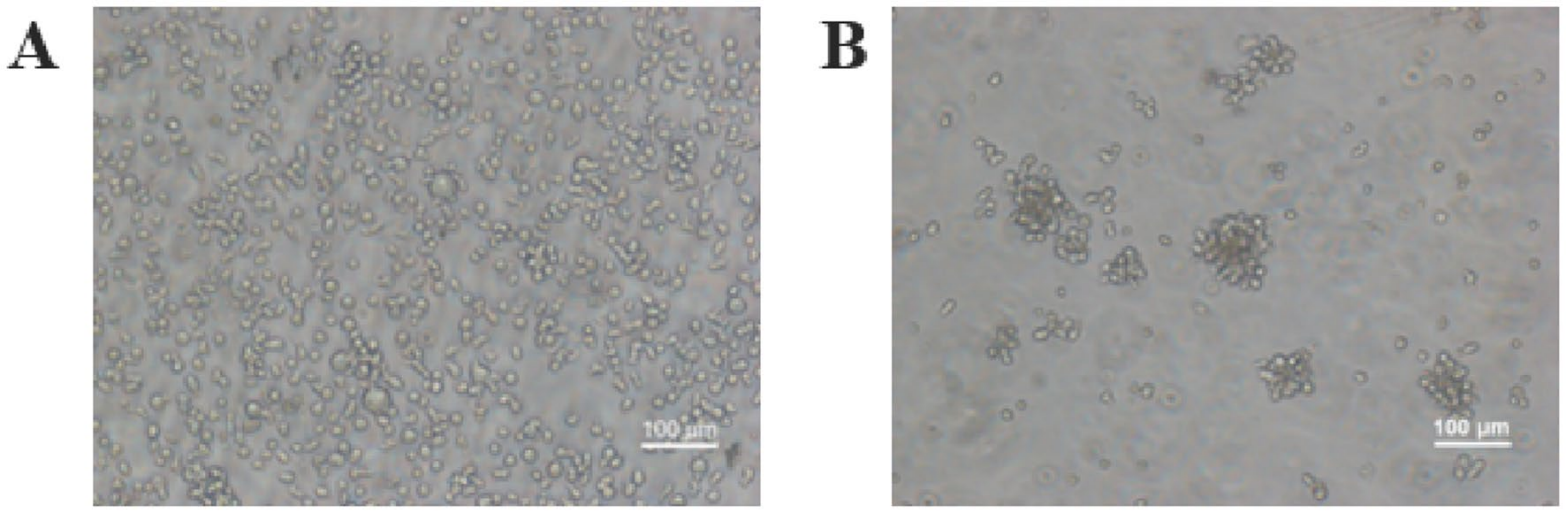
Morphology of NK-92 cells after 8 days of culture. **A** magnetically controlled bioreactor. **B** culture flask

After demonstrating the viability of ex vivo culture of suspension cells, we further evaluated the NK-92 expansion process in the magnetically controlled bioreactor. After 8 days of culture, these cells in the magnetically controlled bioreactor were dispersive and translucent, with clear edges. However, the flask conditions resulted in aggregation and the single cells were small and dim (Fig. 6). The result may be that bioreactors provide a unified environment for cells. In the T25 flask, however, these cells sank to the bottom of the culture flask due to a lack of mixing. The microenvironment around cultured cells is heterogeneous (Curcio et al. 2012; Sadeghi et al. 2011).

**Fig. 7:**
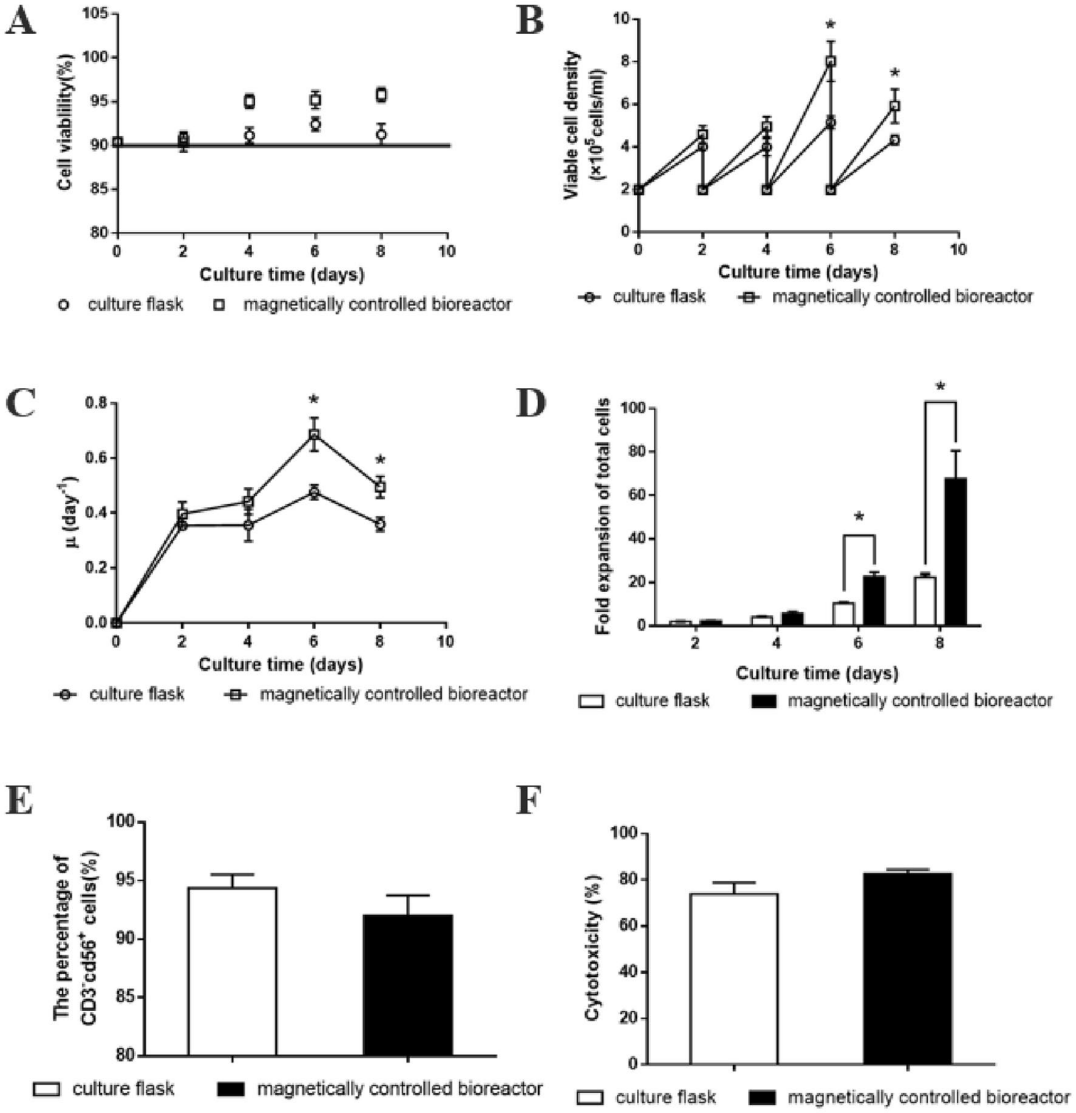
Time profiles of ex vivo expansion and physiological function of NK92 cells in culture flask and the magnetically controlled bioreactor within 8 days (*compared with static cultures, *P* < 0.05). **A** Cell viability. **B** Viable cell density. **C** Specific growth rate of total cells. **D** Expansion folds of total cells. **E** The percentage of CD3^−^CD56^+^ cells of ex vivo-expanded CIK cells. **F** Cytotoxic capacity of ex vivo-expanded CIK cells

Cell viabilities of the magnetically controlled bioreactor with intermittent magnetic fields and T25 culture flask without magnetic fields were both above 90% that during the 8-day culture period (Fig. 7A). And the maximum viable cell densities in the magnetically controlled bioreactor reached 8.04 ± 0.77 × 10^5^ cells/ml, significantly higher than 5.17 ± 0.24 × 10^5^ cells/ml in T25 flask (Fig. 7B). Moreover, the specific growth rate (*μ*) of NK-92 cells was determined based on Eq. (1). The *μ* of NK-92 cells in the magnetically controlled bioreactor and T25 culture flask were increasing gradually from day 0 to 6 but decreased on day 8. Along with the changes of viable cell density, NK-92 cells in the magnetically controlled bioreactor exhibited higher specific growth rate than that in T 25 culture flask (Fig. 7C). Meanwhile, significantly increased expansion was observed after in the magnetically controlled bioreactor (67.71 ± 10.60 folds) and flask (22.41 ± 1.19 folds) cultures (Fig. 7D). To investigate the physiological function of expanded NK-92 cells in bioreactor, cell phenotype and cell killing activity were assessed. CD3^−^CD56^+^ frequencies showed no significant difference between the bioreactor and flask cultures. Similarly, there is no significant difference in the killing activity of the NK-92 cells against the K562 cells between the experimental group and the control group. Taken together, these results indicated that the magnetically controlled bioreactor could improve NK-92 cell expansion without loss of CD3^−^CD56^+^ cells and impairment of cytotoxic capacity (Fig. 7E, F).

Due to the limited number of immune cells, in vitro expansion of cells is required for successful cancer immunotherapy. Conventional culture regimens were mainly performed in static culture with flasks and gas-permeable bags that lack of concern for process parameters, resulting in unstable quality and quantity of cell-products and poor reproducibility. Dynamic suspension culture was a potent method to overcome this disadvantage, however, the growth of immune cells may be impaired as the increasing shear force in the dynamic culture system (Badenes et al. 2016; Liu et al. 2006). In the present study, a magnetically controlled bioreactor using magnetic bead agitation was developed for NK-92 cells expansion. The bioreactor realized homogeneous distribution of the environment through the dynamic magnetic field and magnetic bead. Unlike conventional agitated bioreactors, the agitator of the bioreactor does not require direct contact with the outside environment, avoiding bacterial contamination (Rodling et al. 2018). And that, the agitator of the bioreactor is spherical, which reduces the shearing force of the fluid generated during operation. In addition, intermittent magnetic fields promoted cell expansion while maintaining cell viability and cellular function, though it was not fully deciphered. That might also account for the enhanced amplification of NK cells by the magnetic bioreactor. And it is necessary to explore the mechanism by which the magnetic field affects the cells in the following research. In conclusion, a magnetically controlled bioreactor for ex vivo expansion of NK-92 cells was designed, providing a novel model for expansion of immune cells in the future.

## Conclusions

In this work, a magnetically controlled bioreactor system with a floating magnetic mixer controlled by an intermittent magnetic field was constructed and successfully used to culture NK-92 cells. While maintaining the vitality and function of NK-92 cells, the bioreactor achieved efficient expansion that was superior to the traditional culture flask, showing application prospects in immune cell expansion. In addition, this study is the first report on culturing NK cells in vitro with intermittent magnetic field. In contrast with the static magnetic field, the intermittent magnetic field improved the expansion of NK cells, which provides a viable means for large-scale expansion of NK cells.

## Data Availability

All data generated or analyzed during this study are included in this published article.

## Abbreviations

NK: Natural killer
MHC: Major histocompatibility complex
IL-2: Interleukin-2
GMP: Good manufacturing practices
PBS: Phosphate buffer solution
